# Direct Prosthetic Joint Infection Diagnosis from Sonication Fluid Inoculated in Blood Culture Bottles by Direct MALDI-TOF Mass Spectrometry

**DOI:** 10.3390/diagnostics13050942

**Published:** 2023-03-02

**Authors:** Iñaki Beguiristain, Lucia Henriquez, Ignacio Sancho, Carmen Martin, Angel Hidalgo-Ovejero, Carmen Ezpeleta, Maria Eugenia Portillo

**Affiliations:** 1 Department of Clinical Microbiology, University Hospital of Navarra, 31008 Pamplona, Spain; 2 Institute of Healthcare Research of Navarra (IdiSNa), 31008 Pamplona, Spain; 3 Department of Orthopedics and Trauma Surgery, University Hospital of Navarra, 31008 Pamplona, Spain

**Keywords:** prosthetic joint infection, sonication, MALDI-TOF, blood culture bottles, Sepsityper

## Abstract

An accurate and fast microbiological diagnosis is key for a proper management and results when facing prosthetic joint infection (PJI). The purpose of this study is to assess the role of direct Matrix-assisted laser desorption ionization time of flight (MALDI-TOF) mass spectrometry (MS) for early identification of the pathogens causing PJI from sonication fluid inoculated in blood culture bottles (BCB-SF). This prospective multicentric study included 107 consecutive patients from February 2016 to February 2017. Among them, 71 prosthetic joint revision surgeries were undergone for aseptic and 36 for septic reasons. Prostheses were sonicated and the resulting fluid inoculated into blood culture bottles, regardless the suspicion for infection. We assessed the diagnostic performance of direct MALDI-TOF MS identification of the pathogens in BCB-SF and compared it with periprosthetic tissue and conventional sonication fluid cultures. The sensitivity of direct MALDI-TOF MS of BCB-SF (69%) was higher compared to conventional sonication fluid (69% vs. 64%, *p* > 0.05) or intraoperative tissue cultures (69% vs. 53%, *p* = 0.04), especially for patients receiving antimicrobial treatment. This approach also reduced the time for identification but the specificity was compromised (100% vs. 94%) and polymicrobial infections were missed. In conclusion, BCB-SF improves the sensitivity and reduces the time of PJI diagnosis when used in combination with conventional cultures under strict sterility conditions.

## 1. Introduction

Obtaining culture from periprosthetic tissue samples is the most commonly microbiological methodology applied for the diagnosis of prosthetic joint infections (PJI). However, a considerable rate of infections remain culture negative for numerous reasons, such as incorrect culture media, loss of microbial viability during sample transport, short incubation time, or previous antimicrobial therapy [1,2]. Different microbiological methods have been evaluated for the diagnosis of PJI, such as the sonication of explanted prostheses [1,3,4,5,6], the inoculation of synovial fluid [7], homogenized tissue suspension [8], or the sonication of fluid into aerobic and anaerobic blood culture bottles [9,10,11,12], molecular biology tests [13,14], and nowadays next-generation sequencing methods [15,16]. However, none of the current techniques have shown an adequate diagnostic accuracy to be used as a stand-alone test [17,18]. Rapid identification of the infection microorganisms may aid in choosing an appropriate strategy for revision surgery and targeted antimicrobial therapy.

There are not many studies using MALDI-TOF MS to analyze inoculated BCB with PJI samples to hasten the etiologic diagnosis of this pathology [10,19]. In this study, we evaluated the diagnostic performance of direct MALDI-TOF MS with Sepsityper© specimen processing (Bruker Daltonics, Billerica, MA) for the identification of the pathogens in BCB-SF and compared with periprosthetic tissues and conventional sonication fluid cultures.

The main advantage of this methodology is the speed at which it can provide microbiologic information. By removing a subculture step and directly identifying from clinical samples, the time taken to diagnosis can be further reduced. Direct identification from clinical samples is also possible thanks to molecular biology techniques such as PCR or next-generation sequencing methods [14,20]. However, these technologies are still expensive, require bioinformatics expertise, and are not affordable in every microbiology laboratory, whereas MALDI-TOF MS is currently available in almost all clinical microbiology services [21]. Some studies have recently proven that MALDI-TOF MS can also be used for direct identification of microorganisms without prior culture in certain clinical samples [22].

Here, we investigated whether direct identification from BCB-SF can be implemented for a quick and sensitive tool for the diagnosis of PJI skipping the subculture step. To the best of our knowledge, this is the first study to assess the role of MALDI-TOF MS for direct microbiological identification in positive BCB-SF, applying a simple protein extraction protocol.

## 2. Materials and Methods

**Study design.** A prospective multicentric cohort study was conducted in four tertiary medical care centers, Hospital Universitario de Navarra (≈1000 beds), Hospital Reina Sofia de Tudela (≈200 beds), Hospital García Orcoyen de Estella (≈100 beds), and Clínica Ubarmin covering the entire population of Navarra (≈600,000 inhabitants), in the north of Spain. The ethics committee approved this study, which included the application of an enhanced diagnostic algorithm to all patients to accurately define the cause of prosthesis failure. The protocol included the standardized sampling of three periprosthetic tissue samples, sonication of removed prostheses, prolonged incubation of synovial, periprosthetic tissues, and sonication fluid cultures and inoculation of sonication fluid into aerobic and anaerobic BacT/Alert FAN blood culture bottles with antimicrobial removal systems (BioMérieux, Marcy L’Etoile, France).

**Data sources and participants.** We prospectively included all consecutive patients in the participating centers from February 2016 through February 2017, who underwent a hip or knee prosthesis removal for any reason. Demographic, radiological, medical history, laboratory, and microbiological data, as well as information on type of surgical approach and antimicrobial treatment was recorded.

**Definitions.** PJI was defined according to the definition criteria of the European Bone and Joint Infection Society (EBJIS) [23]. Aseptic failure (AF) was defined when the prosthesis was removed in the absence of these criteria for PJI. Previous antimicrobial treatment was defined as any antimicrobial received for at least 1 day within the previous 14 days of surgery. In all cases, preoperative prophylaxis was administered. 

**Periprosthetic tissue samples.** Tissue samples were intraoperatively collected in native vials. Aliquots of tissue specimens were inoculated in Schaedler enriched with 5% of sheep blood agar plates (BioMérieux, Marcy L’Etoile, France), PoliVitex agar plates (BioMérieux, Marcy L’Etoile, France), and in thioglycollate broth (BBL^TM^ Enriched Thioglycollate Medium with Vitamin K and Hemin, Beckton Dickinson and Company, USA). Aerobic cultures were incubated at 37 °C for one week and anaerobic ones for two weeks. Each distinctive colony morphology was identified by MALDI-TOF MS analysis according to the manufacturer’s instructions. Spectra were obtained and analyzed by the Bruker system.

**Sonication of removed prostheses.** Removed prostheses were explanted in the operating room and transported aseptically to the microbiology laboratory in solid polyethylene airtight containers. Removed prostheses were sonicated in the microbiology laboratory, as previously described by sonication (model SM25E-MT, Branson Ultrasonics Corporation, Geneva, Switzerland) for 1 min (at a frequency of 40 ± 5 kHz) [24]. One milliliter aliquots of sonication fluid was plated onto Schaedler agar plates enriched with 5% sheep blood, PoliVitex chocolate agar plates and inoculated into thioglycollate broth. The cultures were incubated aerobically at 37 °C for 1 week or 2 weeks anaerobically. Positive sonication fluid cultures were considered when ≥50 colony-forming units (CFU) per milliliter of the same organism morphology grew, as previously defined [1,13]. If the patient had previously received antimicrobials, positive sonication fluid culture was defined as growth of ≥1 CFU/mL [25]. Microorganisms grown on culture plates were also submitted to MALDI-TOF MS analysis according to the manufacturer’s instructions. Spectra were obtained and analyzed by the Bruker system.

**Blood culture bottles.** Ten milliliters of sonication fluid was inoculated into aerobic (Plus Aerobic/F culture vials) and anaerobic (Lytic/10 Anaerobic/F culture vials) bottles with antimicrobial removal systems. These bottles were incubated into the automated BD BACTEC FX system (BD Biosciences, Sparks, MD, USA) for up to 5 days. For these bottles which flagged positive, a Gram stain was performed and an inoculum was passaged on a PoliVitex chocolate agar plate, Blood agar plate (BioMérieux, Marcy L’Etoile, France) and Schaedler agar plates enriched with 5% sheep blood as previously described [26]. The aerobic agar plates were incubated 2 more days to complete 1 week, and the anaerobic ones up to complete 2 weeks. 

Microorganisms grown in plates (from sonication fluid and periprosthetic tissue cultures) were identified according to standard methods using MALDI-TOF MS. Positive blood culture bottles (BCB) were analyzed following Sepsityper© analysis protocol. Final microorganisms’ identification using both protocols (direct and grown in plates) was compared.

**Sepsityper© analysis.** One milliliter of sample was collected from BCB was processed using the Sepsityper kit (Bruker) following the manufacturer indications. Subsequent analysis was performed by using the Bruker Microflex LT MALDI-TOF MS and Biotyper 3.0 software. Concisely, 1 mL of residual sonication fluid culture from positive BCB was transferred to a 1.5 mL centrifuge tube. An aliquot of provided lysis buffer was added and the mixture was vortexed and centrifuged. The bacterial pellet was resuspended in the provided wash buffer, vortexed, and centrifuged as before. The pellet was resuspended in 70% ethanol, vortexed, and centrifuged again. The ethanol was discarded and the pellet was allowed to dry completely. When dry, the pellet was resuspended by adding 50 μLof acetonitrile (Sigma-Aldrich). Finally, the suspension was centrifuged once again and 1 μL of the resulting supernatant was analyzed using MALDI-TOF MS.

**MALDI-TOF MS Analysis**. Two microliters of aliquot for each extracted protein supernatant was placed in individual spots on the target plate (Bruker) and allowed to dry. Each spot was covered with 1 μLof alpha-cyano-4-hydroxy cinamic acid (HCCA) matrix and allowed to dry. When dry, the target plate was inserted into the Bruker Microflex LT MALDI-TOF MS system for analysis. A profile of proteins with *m*/*z* of 3000 to 15000 was generated for each specimen and analyzed using Biotyper 3.0 software (Bruker). The top 10 identification matches along with confidence scores within 0.0 to 3.0 were ordered by the system. Scores ≥2.0 were considered as high-confidence identification (secure species), scores of 1.7 to 1.99 as intermediate-confidence identification (secure genus only), and scores <1.7 as unacceptable identification. 

**Statistical Analysis**. Statistical significance was calculated using McNemar’s chi-squared test (two-sided) for sensitivity comparison. Probability (*p*) values of less than 0.05 were considered statistically significant. Calculations and graphics were performed using Prism software (Version 6.05; GraphPad, La Jolla, CA, USA).

## 3. Results

### 3.1. Study Population

**Figure 1:**
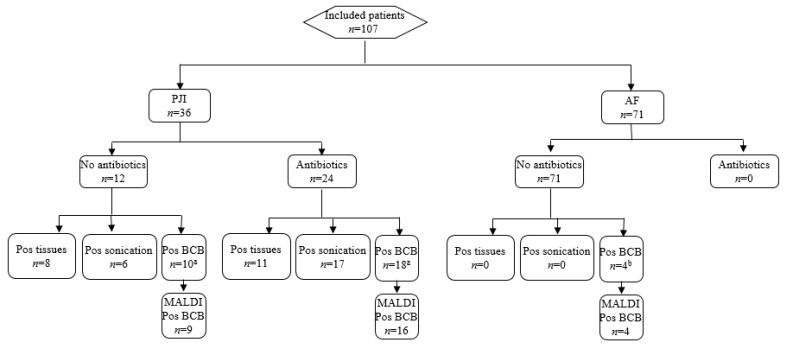
Concordance of sonication fluid cultures, periprosthetic tissue cultures and direct MALDI-TOF MS of positive blood culture bottles. Note: PJI, prosthetic joint infection; AF, aseptic failure; Pos, positive; Neg, negative; BCB, blood culture bottles. ^a^ Two cases (3%) had growth of low-virulent microorganisms below the cut-off (50 CFU/mL); ^b^ Two coagulase negative staphylococci (CNS) and two *Cutibacterium acnes*.

We included 107 patients in whom a prosthetic implant was removed. No patients were excluded due to obvious contaminations of the implant. AF was diagnosed in 71 cases (66%) and PJI in 36 cases (34%). Two thirds of patients with PJI (67%) received antimicrobials within 14 days prior sampling. The results summary is shown in Figure 1.

### 3.2. MALDI-TOF MS Identification

A total of 36 PJI were analyzed. Table 1 shows the microbiological findings in these infection cases.

Of 17 (47%) monomicrobial cultures, 71% contained Gram-positive microorganisms, 14% contained Gram-negative microorganisms, 10% contained *C. acnes* and 5% yeasts. Two (6%) cultures were polymicrobial and the remaining 47% were negative periprosthetic tissue cultures. The rate of negative culture decreased from 47% by using periprosthetic tissue cultures to 36% and 31% by using conventional sonication fluid and BCB-SF, respectively.

Four microorganisms which grew in conventional cultures were not identified by MALDI-TOF MS of the positive BCB-SF. This included 1 *C. acnes*, 1 *E. faecium* and 2 microorganisms related to polymicrobial infections (1 *E.hermanii* and 1 coagulase negative Staphylococci, CNS). The MALDI-TOF MS correctly identified only one of the microorganisms present in the polymicrobial infections. Gram-negative bacteria were more likely to produce high confidence scores than Gram-positive bacteria. Overall concordance with classical identification techniques was 100% to genus and species for isolates with scores of ≥1.7.There were no discrepant identification results, with scores <1.7 either. *Candida* spp. could not be identified by MALDI-TOF MS because it was also not isolated from conventional sonication fluid culture. 

### 3.3. Comparison of Diagnostic Methods 

Table 2 summarizes the culture accuracy of periprosthetic tissue, conventional sonication fluid, and MALDI-TOF MS of BCB-SF from patients with PJI and AF.

The sensitivity of BCB-SF was significantly higher than that of periprosthetic tissue culture (78% vs. 53%, *p* = 0.003). BCB-SF direct MALDI-TOF MS identification’s sensitivity was slightly lower but remained significantly higher compared to periprosthetic tissue culture (69% vs. 53%, *p* = 0.04). However, the specificity of this technique was lower than periprosthetic tissue and sonication fluid culture (94% vs. 100%)

### 3.4. Effect of Previous Antibiotic Treatment

**Figure 2:**
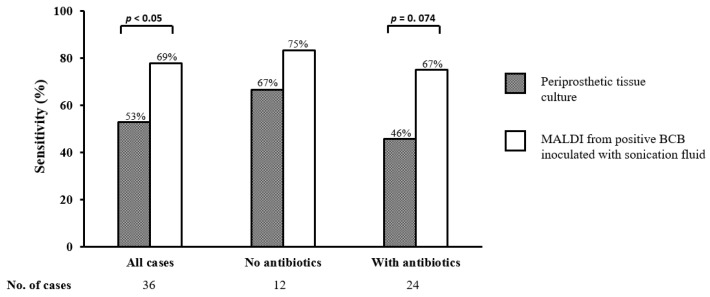
Sensitivity of periprosthetic tissue culture and direct MALDI-TOF MS from sonication fluid inoculated into blood culture bottles in 36 prosthetic joint infections (stratified to whether or not receiving antibiotics prior to sampling).

Figure 2 shows the sensitivity of periprosthetic tissue cultures and MALDI-TOF MS of positive BCB-SF. 

Previous antibiotic therapy reduced the sensitivity of periprosthetic tissue cultures from 67% to 46%, and the sensitivity of MALDI-TOF MS of positive BCB-SF from 75% to 67%. Among the 71 cases with AF, none received antimicrobial treatment prior to surgery and all cases were negative by conventional periprosthetic tissue and sonication fluid cultures. In four cases of AF, direct MALDI-TOF MS of BCB-SF detected two CNS and two *C. acnes*. None of these low virulent microorganisms grew in conventional sonicate fluid cultures. The rapid identification with MALDI-TOF MS of positive BCB-SF showed a significantly superior sensitivity compared to standard periprosthetic tissue cultures (*p* = 0.04). In patients receiving antibiotics prior to surgery, this novel methodology improved the sensitivity from 46% with periprosthetic tissue cultures to 67%; however, this difference was not statistically significant due to the small sample size.

### 3.5. Microbiological Findings

Table 1 shows the microbiological findings of individual diagnostic techniques in 36 patients with PJI. Using conventional periprosthetic tissue cultures, fewer pathogens were detected (n = 21) than by conventional and MALDI-TOF MS of positive BCB-SF (n = 25). Polymicrobial infections (i.e., isolation of ≥2 microorganisms) were detected by periprosthetic tissue cultures or conventional sonication fluid cultures but not by the direct MALDI-TOF MS of positive BCB-SF. All patients with negative cultures received antibiotics prior to surgery. 

### 3.6. Time to Identification

**Figure 3:**
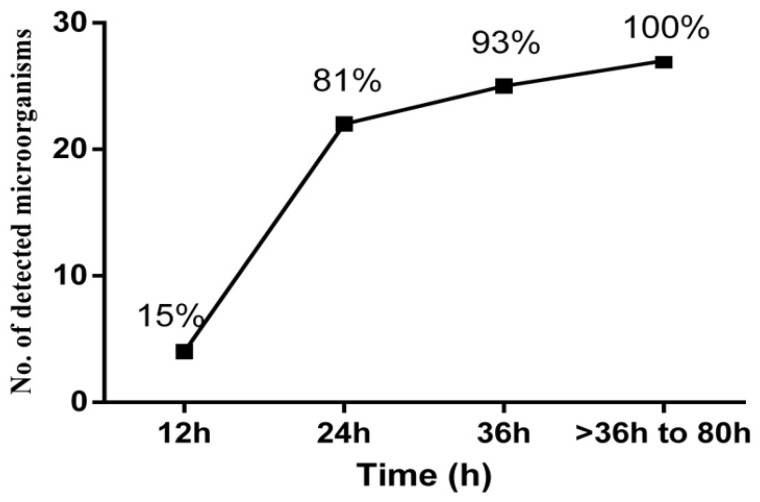
Time to positivity of positive blood culture bottles inoculated with sonication fluid.

In most of the positive BCB-SF, bacterial growth was detected in the first 24 h. Time to positivity of these cultures is shown in Figure 3. 

Due to the BCB inoculation, microbial growth was detected in 15% and 81% of the positive samples in the first 12 h and 24 h after surgery respectively.

**Figure 4:**
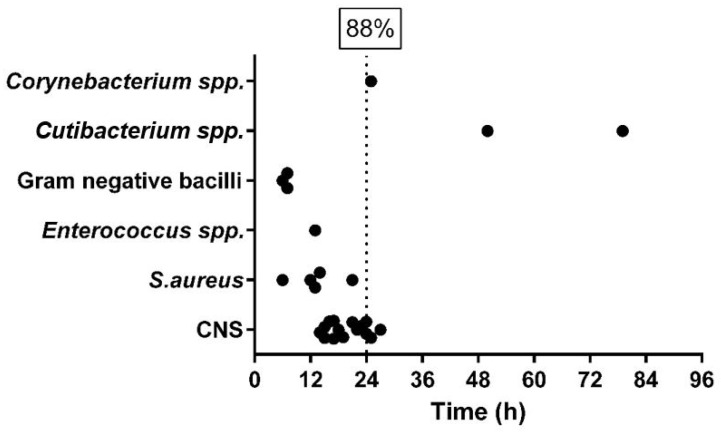
Direct turnaround time for direct pathogen identification by MALDI-TOF MS of positive BCB with sonication fluid (stratified to the type of pathogen).

Figure 4 Illustrates the time to identification by direct MALDI-TOF MS from positive BCB-SF with sonication fluid.

Gram-negative bacteria required the shortest times (less than 12 h of incubation). After one day of incubation, 88% of PJI were already diagnosed. In this series, all pathogens except *C. acnes* were identified in the first 36h of incubation.

## 4. Discussion

The inoculation of synovial fluid and homogenized periprosthetic tissue into aerobic and anaerobic BCB improves the sensitivity of culture for PJI diagnosis [7,9]. Later, some researchers proved improved sensitivity of sonication fluid culture compared to periprosthetic tissue cultures [5,6,24,27]. In a previous publication, the inoculation of sonication fluid into BCB has been compared with the inoculation of synovial fluid [11]. However, it remains unclear whether the inoculation of sonication fluid into BCB increases sensitivity compared to conventional sonication fluid cultures because the same sample (in this case sonication fluid) was not compared in that study. 

In this study, we compared the performance of BCB-SF with periprosthetic tissue and sonication fluid cultures. When sonication fluid was inoculated into BCB and microbial identification was performed by direct MALDI-TOF MS, the sensitivity was higher (69%), compared to conventional sonication fluid (64%), and periprosthetic tissue samples (53%). Despite the fact that sonication may cause damage to microorganisms, especially to Gram-negative bacilli and anaerobic bacteria [28], the same number of these microorganisms isolated by tissues were detected by conventional sonication, corroborating the efficacy of sonication procedure. 

In the study by Shen et al. [11], researchers detected six additional infections by BCB-inoculated sonication fluid compared to synovial fluid in patients with previous antibiotic treatment. CNS were isolated from some BCB inoculated with sonication fluid from patients with AF, which may reflect either the contamination or misclassification of patients.

In order to correctly interpret the BCB cultures, it is crucial to perform other conventional cultures and consider them together with other evidence [23]. In patients for whom the infection is unlikely and both periprosthetic tissues and sonication fluid cultures are negative, positive BCB cultures must be interpreted as contaminants. The inoculation of sonication fluid in BCB has several advantages when used in combination with conventional solid media cultures. Previous studies have suggested that culture-negative cases of PJI microorganisms may be present in the sonication fluid (or at least their DNA), as detected by broad-range PCR [29] or multiplex PCR [13,30]. Nevertheless, it is not yet clear if culture negativity is caused by a low microbial load, under the detection limit of conventional sonication fluid cultures, or by non-viability of the pathogens. By using a blood culture system, a 20-fold increased volume of sonication fluid is inoculated compared to a solid media plate culture (10 mL vs. 0.5 mL). Our study also suggests that more sample volume increases the sensitivity but compromises the culture specificity. Consequently, it is crucial to perform the inoculation under strict sterile conditions and to rely on a microbiologic method based on more than one culture. In addition, growth media in BCB contain antimicrobial removal systems and allow growth of microorganisms immediately after inoculation. The main disadvantage in the inoculation of BCB-SF is a potential decrease in specificity due to the loss of colony count thresholds defining a positive culture. Other authors have pointed out that sonication fluid inoculation in BCB decreases the sensitivity for anaerobic microorganisms [31], which is in line with our findings. Consequently, a combination of independent microbiologic diagnostic tests should be performed, including periprosthetic tissue cultures and conventional sonication fluid cultures.

In our study, previous antibiotic therapy reduced the culture sensitivity of all culture diagnostics methods. However, the sensitivity of BCB-SF was higher in patients receiving antibiotics prior to surgery (67% vs. 46%). Therefore, the inoculation of BCB-SF with antimicrobial removal systems may be principally useful in this group of patients. 

It has been described that sonication fluid culture increases sensitivity in chronic, but not in acute PJI [24]. Interestingly, by the inoculation of BCB-SF, three additional cases of PJI were detected in our study (and not by conventional sonication fluid culture). These three cases were acute infections, receiving previous antibiotics, and this may reflect that BCB-SF inoculation is advisable for the diagnosis of both acute and chronic PJI. In acute infections, antibiotic therapy is usually initiated before surgery, reducing the efficiency of traditional cultures. In the case of chronic infections, where low-virulence microorganisms are involved and biofilms are mature and more firmly close to the prosthesis surface, sonication can fully exhibit its detachment effect and improved detection characteristics.

Despite the use of sonication improving the diagnosis of PJI, a significant number of these infections remain culture negative [1,25,30,32]. Culture-negative PJI may be due to different causes as the use of prior antimicrobial therapy, inappropriate diagnostic methods, or because of the pathogen’s inability to grow with currently available culture methods [24,33,34]. Different non-culture techniques, such as PCR or next-generation sequencing (NGS), are now available [14]. NGS has different approaches, allowing sequencing of the whole genome of the pathogen to detecting all the genomes present in a clinical sample. Currently, many knowledge gaps related to NGS have been identified, so more comparative studies with clear methodology are needed in order to reach conclusions [35]. Moreover, the high cost, the need for specific equipment, and for microbiologists specialized in bioinformatics makes NGS only available in reference laboratories.

In our study, direct MALDI-TOF MS from positive BCB-SF succeeded in significantly reducing the rate of culture-negative PJI from 47% (by periprosthetic tissue culture) to 31% (*p* = 0.04). We also observed that the time to microbial detection was further reduced by inoculating BCB-SF, detecting an overall of 81% of infections and identifying 88% of these within 24 h of culture incubation. That means that by applying direct MALDI-TOF MS from positive BCB-SF, we can have the pathogen identification within the first 2 days after surgery except if the causative microorganism is *C. acnes*. These results are in agreement with prior studies, which demonstrated that sonication fluid provided faster microbial detection than periprosthetic tissue samples [1]. Therefore, direct MALDI-TOF MS from positive BCB-SF is an approach that is cheap and easy to implement in any laboratory that improves the sensitivity and reduces the time of PJI diagnosis. Moreover, this approach may help to provide early guidance for targeted antibiotic therapy and even determine whether antimicrobial stewardship should be implemented in cases of negative results [36].

## 5. Conclusions

Direct MALDI-TOF MS from positive BCB-SF with antimicrobial removal systems improved the diagnosis of PJI when used in combination with conventional sonication fluid culture. This approach considerably reduced the time of PJI identification compared to conventional sonication fluid and periprosthetic tissue cultures. This method demonstrated high rates of sensitivity and specificity, especially in patients receiving antibiotics prior to surgery. This straightforward and affordable diagnostic method may significantly improve the diagnosis of implant-associated infections in laboratories where molecular biology methods are not available. Although rapid identification results by direct MALDI-TOF MS seem encouraging, subculturing still remains necessary when the direct analysis fails to achieve species identification in the case of a polymicrobial infection. 

Finally, we conclude that direct identification by MALDI-TOF MS of positive BCB-SF by applying a simple protocol for protein extraction is an agile and promising approach for clinical laboratories to improve and hasten the etiologic diagnosis of PJI when used in combination with other culture-based methods.

## Figures and Tables

**Table 1:** Microbiological findings in 36 PJI cases according to type of diagnostic method.

Characteristics	Periprosthetic Tissue Culture	Conventional Sonication Fluid Culture	MALDI-TOF of Sonication Fluid Inoculated in Blood Culture Bottles
No. of detected PJI			
Monomicrobial	17 (47%)	21 (58%)	25 (69%)
Polymicrobial	2 (6%) ^a^	2 (6%) ^a^	0 (0%)
Negative culture	17 (47%)	13 (36%)	11 (31%)
Total No. of microorganisms isolated	21	25	25
Gram-positive bacteria	15 (71%)	20 (80%)	22 (88%)
Coagulase negative staphylococci	8	13	16
*S. aureus*	5	5	5
*Enterococcus* spp.	1	1	0
*Corynebacterium* spp.	1	1	1
Gram-negative bacteria	3 (14%)	3 (12%)	2 (8%)
*Escherichia* spp.	2	2	1
*Enterobacter* spp.	1	1	1
Anaerobes	2 (10%)	2 (8%)	1 (4%)
*C. acnes*	2	2	1
Yeasts	1 (5%)	0 (0%)	0 (0%)
*Candida* spp.	1	0	0

^a^ S. epidermidis + S. caprae; E. cloacae + E. hermannii.

**Table 2:** Diagnostic performance of three diagnostic methods in 107 patients with removed prosthesis (36 with prosthetic joint infection and 71 with aseptic failure).

Diagnostic Method	Sensitivity, % (95% CI)	Specificity, % (95% CI)	PPV, % (95% CI)	NPV, % (95% CI)
Periprosthetic tissue samples	53 (35–70)	100 (100–100)	100 (100–100)	81 (71–88)
Conventional sonication fluid	64 (46–79)	100 (100–100)	100 (100–100)	85 (75–91)
Sonication fluid in blood culture bottles	78 (61–90)	94 (89–98)	88 (71–96)	89 (80–95)
MALDI-TOF MS of sonication fluid in blood culture bottles	69 (52–84)	94 (89–98)	86 (68–96)	86 (76–93)

Note: CI, confidence interval; PPV, positive predictive value; NPV, negative predictive value.

## Data Availability

Not applicable.

## References

[1] Portillo M.E., Salvado M., Alier A., Martinez S., Sorli L., Horcajada J.P.P., Puig L., Salvadó M., Alier A., Martínez S. (2014). Advantages of sonication fluid culture for the diagnosis of prosthetic joint infection. J. Infect..

[2] Esteban J. (2022). Microbiologial diagnosis of prosthetic joint infection: Is there a need for standardization?. Enferm. Infecc. Microbiol. Clin. (Engl. Ed.).

[3] Trampuz A., Piper K.E., Hanssen A.D., Osmon D.R., Cockerill F.R., Steckelberg J.M., Patel R. (2006). Sonication of explanted prosthetic components in bags for diagnosis of prosthetic joint infection is associated with risk of contamination. J. Clin. Microbiol..

[4] Portillo M.E., Corvec S., Borens O., Trampuz A. (2013). Propionibacterium acnes: An underestimated pathogen in implant-associated infections. Biomed. Res. Int..

[5] Holinka J., Bauer L., Hirschl A.M., Graninger W., Windhager R., Presterl E. (2011). Sonication cultures of explanted components as an add-on test to routinely conducted microbiological diagnostics improve pathogen detection. J. Orthop. Res..

[6] Piper K.E., Jacobson M.J., Cofield R.H., Sperling J.W., Sanchez-Sotelo J., Osmon D.R., McDowell A., Patrick S., Steckelberg J.M., Mandrekar J.N. (2009). Microbiologic diagnosis of prosthetic shoulder infection by use of implant sonication. J. Clin. Microbiol..

[7] Font-Vizcarra L., Garcia S., Martinez-Pastor J.C., Sierra J.M., Soriano A. (2010). Blood culture flasks for culturing synovial fluid in prosthetic joint infections. Clin. Orthop. Relat. Res..

[8] Lallemand E., Arvieux C., Coiffier G., Polard J.L., Albert J.D., Guggenbuhl P., Jolivet-Gougeon A. (2017). Use of MALDI-TOF mass spectrometry after liquid enrichment (BD Bactec^TM^) for rapid diagnosis of bone and joint infections. Res. Microbiol..

[9] Velay A., Schramm F., Gaudias J., Jaulhac B., Riegel P. (2010). Culture with BACTEC Peds Plus bottle compared with conventional media for the detection of bacteria in tissue samples from orthopedic surgery. Diagn. Microbiol. Infect. Dis..

[10] Minassian A.M., Newnham R., Kalimeris E., Bejon P., Atkins B.L., Bowler I.C. (2014). Use of an automated blood culture system (BD BACTEC) for diagnosis of prosthetic joint infections: Easy and fast. BMC Infect. Dis..

[11] Shen H., Tang J., Wang Q., Jiang Y., Zhang X. (2014). Sonication of Explanted Prosthesis Combined with Incubation into BD BACTEC Bottles for Pathogen Diagnosis of Prosthetic Joint Infection. J. Clin. Microbiol..

[12] Janz V., Wassilew G.I., Hasart O., Matziolis G., Tohtz S., Perka C. (2013). Evaluation of sonicate fluid cultures in comparison to histological analysis of the periprosthetic membrane for the detection of periprosthetic joint infection. Int. Orthop..

[13] Achermann Y., Vogt M., Leimig M., Wüst J., Trampuz A., Leunig M., Wust J., Trampuz A. (2010). Improved diagnosis of periprosthetic joint infection by multiplex PCR of sonication fluid from removed implants. J. Clin. Microbiol..

[14] Esteban J., Gómez-Barrena E. (2021). An update about molecular biology techniques to detect orthopaedic implant-related infections. EFORT Open Rev..

[15] Gatti G., Taddei F., Brandolini M., Mancini A., Denicolò A., Congestrì F., Manera M., Arfilli V., Battisti A., Zannoli S. (2022). Molecular Approach for the Laboratory Diagnosis of Periprosthetic Joint Infections. Microorganisms.

[16] Street T.L., Sanderson N.D., Kolenda C., Kavanagh J., Pickford H., Hoosdally S., Cregan J., Taunt C., Jones E., Oakley S. (2022). Clinical Metagenomic Sequencing for Species Identification and Antimicrobial Resistance Prediction in Orthopedic Device Infection. J. Clin. Microbiol..

[17] Corvec S., Portillo M.E., Pasticci B.M.B.M., Borens O., Trampuz A. (2012). Epidemiology and new developments in the diagnosis of prosthetic joint infection. Int. J. Artif. Organs.

[18] Zimmerli W., Trampuz A., Ochsner P.E. (2004). Prosthetic-joint infections. N. Engl. J. Med..

[19] Kuo F.C., Chien C.C., Lee M.S., Wang J.W., Lin P.C., Lee C.H. (2020). Rapid diagnosis of periprosthetic joint infection from synovial fluid in blood culture bottles by direct matrix-assisted laser desorption ionization time-of-flight mass spectrometry. PLoS ONE.

[20] Street T.L., Sanderson N.D., Atkins B.L., Brent A.J., Cole K., Foster D., McNally M.A., Oakley S., Peto L., Taylor A. (2017). Molecular diagnosis of orthopedic-device-related infection directly from sonication fluid by metagenomic sequencing. J. Clin. Microbiol..

[21] Schubert S., Kostrzewa M. (2017). MALDI-TOF MS in the Microbiology Laboratory: Current Trends. Curr. Issues Mol. Biol..

[22] Tsuchida S., Umemura H., Nakayama T. (2020). Current Status of Matrix-Assisted Laser Desorption/Ionization-Time-of-Flight Mass Spectrometry (MALDI-TOF MS) in Clinical Diagnostic Microbiology. Molecules.

[23] McNally M., Sousa R., Wouthuyzen-Bakker M., Chen A.F., Soriano A., Vogely H.C., Clauss M., Higuera C.A., Trebse R. (2021). The EBJIS definition of periprosthetic joint infection: A practical guide for clinicians. Bone Jt. J..

[24] Trampuz A., Piper K.E., Jacobson M.J., Hanssen A.D., Unni K.K., Osmon D.R., Mandrekar J.N., Cockerill F.R., Steckelberg J.M., Greenleaf J.F. (2007). Sonication of removed hip and knee prostheses for diagnosis of infection. N. Engl. J. Med..

[25] Portillo M.E., Salvado M., Trampuz A., Plasencia V., Rodriguez-Villasante M., Sorli L., Puig L., Horcajada J.P.P., Salvadó M., Trampuz A. (2013). Sonication versus vortexing of implants for diagnosis of prosthetic joint infection. J. Clin. Microbiol..

[26] Portillo M.E., Salvadó M., Trampuz A., Siverio A., Alier A., Sorli L., Martínez S., Pérez-Prieto D., Horcajada J.P.J.P., Puig-Verdie L. (2015). Improved diagnosis of orthopedic implant-associated infection by inoculation of sonication fluid into blood culture bottles. J. Clin. Microbiol..

[27] Tande A.J., Patel R. (2014). Prosthetic joint infection. Clin. Microbiol. Rev..

[28] Monsen T., Lovgren E., Widerstrom M., Wallinder L. (2009). In vitro effect of ultrasound on bacteria and suggested protocol for sonication and diagnosis of prosthetic infections. J. Clin. Microbiol..

[29] Gomez E., Cazanave C., Cunningham S.A., Greenwood-Quaintance K.E., Steckelberg J.M., Uhl J.R., Hanssen A.D., Karau M.J., Schmidt S.M., Osmon D.R. (2012). Prosthetic joint infection diagnosis using broad-range PCR of biofilms dislodged from knee and hip arthroplasty surfaces using sonication. J. Clin. Microbiol..

[30] Portillo M.E., Salvado M., Sorli L., Alier A., Martinez S., Trampuz A., Gomez J., Puig L., Horcajada J.P. (2012). Multiplex PCR of sonication fluid accurately differentiates between prosthetic joint infection and aseptic failure. J. Infect..

[31] Rieber H., Frontzek A., Alefeld M., Heinrich S., Barden B., Jerosch J., Breil-Wirth A., Schmitt H., Ulatowski M., Götz S. (2020). Sonicate fluid inoculated into blood culture bottles does not improve diagnosis of periprosthetic joint infection caused by anaerobes. A retrospective analysis. Anaerobe.

[32] Berbari E.F., Marculescu C., Sia I., Lahr B.D., Hanssen A.D., Steckelberg J.M., Gullerud R., Osmon D.R. (2007). Culture-negative prosthetic joint infection. Clin. Infect. Dis..

[33] Cazanave C., Greenwood-Quaintance K.E., Hanssen A.D., Karau M.J., Schmidt S.M., Gomez Urena E.O., Mandrekar J.N., Osmon D.R., Lough L.E., Pritt B.S. (2013). Rapid molecular microbiologic diagnosis of prosthetic joint infection. J. Clin. Microbiol..

[34] Tande A.J., Cunningham S.A., Raoult D., Sim F.H., Berbari E.F., Patel R. (2013). A case of Q fever prosthetic joint infection and description of an assay for detection of Coxiella burnetii. J. Clin. Microbiol..

[35] Portillo M.E., Sancho I. (2023). Advances in the Microbiological Diagnosis of Prosthetic Joint Infections. Diagnostics.

[36] Vidal P., Fourniols E., Junot H., Meloni C., Bleibtreu A., Aubry A. (2022). Antibiotic Stewardship in Treatment of Osteoarticular Infections Based on Local Epidemiology and Bacterial Growth Times. Microbiol. Spectr..

